# Statistical approaches to identify subgroups in meta-analysis of individual participant data: a simulation study

**DOI:** 10.1186/s12874-019-0817-6

**Published:** 2019-09-02

**Authors:** Michail Belias, Maroeska M. Rovers, Johannes B. Reitsma, Thomas P. A. Debray, Joanna IntHout

**Affiliations:** 1 Radboud Institute for Health Sciences (RIHS), Radboud university medical center, Mailbox 133, P.O. Box 9101, Nijmegen, 6500 HB The Netherlands; 2 Julius Center for Health Sciences and Primary Care, University Medical Center Utrecht, Utrecht University, PO Box 85500, 3508 GA Utrecht, The Netherlands; 3 Cochrane Netherlands, University Medical Center Utrecht, Utrecht University, PO Box 85500, Utrecht, 3508 GA The Netherlands

**Keywords:** Subgroups, Simulation, Individual participant data, Effect modification, Meta-analysis, Statistical approaches, Comparison, Ecological bias, Heterogeneity

## Abstract

**Background:**

Individual participant data meta-analysis (IPD-MA) is considered the gold standard for investigating subgroup effects. Frequently used regression-based approaches to detect subgroups in IPD-MA are: meta-regression, per-subgroup meta-analysis (PS-MA), meta-analysis of interaction terms (MA-IT), naive one-stage IPD-MA (ignoring potential study-level confounding), and centred one-stage IPD-MA (accounting for potential study-level confounding). Clear guidance on the analyses is lacking and clinical researchers may use approaches with suboptimal efficiency to investigate subgroup effects in an IPD setting. Therefore, our aim is to overview and compare the aforementioned methods, and provide recommendations over which should be preferred.

**Methods:**

We conducted a simulation study where we generated IPD of randomised trials and varied the magnitude of subgroup effect (0, 25, 50% relative reduction), between-study treatment effect heterogeneity (none, medium, large), ecological bias (none, quantitative, qualitative), sample size (50,100,200), and number of trials (5,10) for binary, continuous and time-to-event outcomes. For each scenario, we assessed the power, false positive rate (FPR) and bias of aforementioned five approaches.

**Results:**

Naive and centred IPD-MA yielded the highest power, whilst preserving acceptable FPR around the nominal 5% in all scenarios. Centred IPD-MA showed slightly less biased estimates than naïve IPD-MA. Similar results were obtained for MA-IT, except when analysing binary outcomes (where it yielded less power and FPR < 5%). PS-MA showed similar power as MA-IT in non-heterogeneous scenarios, but power collapsed as heterogeneity increased, and decreased even more in the presence of ecological bias. PS-MA suffered from too high FPRs in non-heterogeneous settings and showed biased estimates in all scenarios. Meta-regression showed poor power (< 20%) in all scenarios and completely biased results in settings with qualitative ecological bias.

**Conclusions:**

Our results indicate that subgroup detection in IPD-MA requires careful modelling. Naive and centred IPD-MA performed equally well, but due to less bias of the estimates in the presence of ecological bias, we recommend the latter.

**Electronic supplementary material:**

The online version of this article (10.1186/s12874-019-0817-6) contains supplementary material, which is available to authorized users.

## Introduction

Meta-analyses of individual participant data (IPD) provide the best evidence regarding treatment effects and offer unique opportunities and benefits when investigating subgroup effects [1]. Therefore, IPD meta-analyses (IPD-MA) are considered the gold standard for detection of subgroup effects and their use has increased over the last decade [2, 3]. The possibility to standardize subgroup definitions and outcomes across studies, the higher validity and credibility of subgroup findings and the increased flexibility to search for subgroups based on combinations of patient and/or disease characteristics and the avoidance of incorrect results due to ecological bias are benefits of using IPD of multiple trials rather than traditional (aggregate) meta-analysis [4]. Nevertheless, Simmonds et al. (2015) have reported that only 1% of the conducted meta-analyses were using IPD [5].

IPD-MA may be conducted either in one or two stages. In two-stage IPD-MA, each trial is first analysed separately, using an appropriate statistical model. For instance, the first stage may estimate the main treatment effect, or the different effects observed per subgroup, or the treatment-covariate interaction effect. Subsequently, these effects from different trials are combined into a summary estimate in the second stage of the meta-analysis. Although meta-analytic methods are often used to investigate main treatment effects, they can also be used to investigate subgroups. For instance, the presence of subgroup effects can be investigated by modelling the association of the estimated main treatment effects with a trial-level covariate (meta-regression). Alternatively, estimates of subgroup effects or interaction terms can directly be summarized using traditional meta-analysis (MA) methods.

In one-stage IPD-MA, all IPD from every trial are analysed simultaneously whilst accounting for the clustering of participants within studies. Hereby, researchers may model interactions between treatment and patient-level covariates either directly (naive IPD-MA), or after the covariates are mean-centred per study in order to account for potential ecological bias (centred IPD-MA) [6].

When IPD are available for all studies, it is often unclear which meta-analysis method should be adopted. In 2015, Simmonds et al. reported that all approaches are still being used in IPD-MA, even aggregated data meta-analytic methods such as meta-regression and PS-MA. Although minor differences are usually observed when summarizing main treatment effects, each of aforementioned methods have specific deficiencies when investigating the presence of subgroup effects [7, 8]. In particular, it is well known that meta-regression has poor power and is prone to (ecological) bias [6, 9–11]. Per-subgroup MA (PS-MA) has also been criticized of being prone to ecological bias [12]. Further, MA of interaction terms (MA-IT) is considered as less precise when limited number of studies or participants are present [2]. Finally, it has been demonstrated that “naive” IPD-MA may suffer from limited precision and excessive false positive rates (type I error) in the presence of trial-level confounding [6], which is similar to ecological bias.

So far, comparisons of meta-regression, PS-MA, MA-IT, “naïve” and centred meta-analysis to study subgroup effects have been limited to either empirical studies or simulation studies only comparing a subset of these approaches. Simmonds and Higgins [13] proved that one-stage IPD-MA is always more powerful than the two-stage methods, under the assumption that there is no between-study heterogeneity, all studies have the same residual variance and all studies use balanced randomization. These assumptions were considered too restrictive. Therefore, Simmonds and Higgins also performed a simulation to compare meta-regression and MA-IT, but their simulations only included datasets with 250 patients and neither one-stage methods nor PS-MA were included. Other studies ignored the presence of residual (i.e. unrelated to effect modification) between-study heterogeneity in treatment effect, for example Lambert et al. [10] compared meta-regression to naïve IPD-MA using simulated datasets without between-study heterogeneity. Koopman et al. compared meta-regression, naive IPD-MA, and MA-IT using only empirical studies [14]. Hua et al. compared different types of one-stage approaches using simulated time-to-event data [6]. Burke et al. theoretically explained the differences between the results of naive IPD-MA and MA-IT [7]. Fisher et al. wrote a critical review over PS-MA, MA-IT, centred and naive IPD-MA methods and applied them on empirical studies [8]. In a subsequent paper, comparing two-stage methods only, he advocated the use of MA-IT over PS-MA and meta-regression, and applied all three on empirical studies to point out the differences [12]. Simmonds et al. [5, 15] reviewed the aforementioned statistical approaches in two consecutive papers, one in 2005 and one in 2015. They concluded that one-stage methods are used more frequently in 2015 than in 2005 and that meta-regression and MA-IT were not typically preferred, in contrast to PS-MA which is the most frequently used two-stage method. PS-MA, however, is prone to power reduction when heterogeneity is present and overestimation when there is no heterogeneity [12, 16, 17]. These issues may be worsened if ecological bias and between-study heterogeneity are simultaneously present. Finally, Kontopantelis has performed a simulation study comparing naïve IPD-MA and MA-IT [18]. He has generated data, covering different IPD sizes and different between-study heterogeneity levels on intercept and treatment effect. Nevertheless, centred IPD-MA, PS-MA, and meta-regression were not included, ecological bias was not generated and the number of participants was above 1000, which in some RCTs may be unrealistic.

To our knowledge all above mentioned studies have focused on a limited number of scenarios. Most studies either focused on only a limited set of available approaches, only one type of outcome or one level of subgroup effect magnitude and only in Hua et al. [6] ecological bias has been introduced.

In our simulation paper, we will compare all aforementioned approaches using binary, continuous and survival outcome measures, focussing on differences in power, false positive rates (FPRs) and bias in the estimates. We will vary the amount of between-study heterogeneity in treatment effects, the magnitude of the subgroup effect, the level of ecological bias, and the number of trials and participants.

## Methods

In our study we included five common statistical approaches: centred (one-stage) IPD-MA, naive (one-stage) IPD-MA, MA of interaction terms (MA-IT), per-subgroup MA (PS-MA), and meta-regression. We simulated datasets with binary, continuous, or survival outcomes. We varied the magnitude of the subgroup effects, the presence of between-study heterogeneity in treatment effect, the level of ecological bias, and the size and number of trials. Our paper is organized as follows. We start with a description of the data generation mechanism (Section 2.1), followed by a description of the statistical approaches (Section 2.2), and the assessment of power, FPRs and bias in the estimates (Section 2.3).

### Data generation

In this section we describe the data-generation mechanism in general; details on the parameters can be found in Table 1. In short, IPD-sets were generated for continuous, binary, and time-to-event outcomes. We generated equal treatment allocation, as in a two-arm randomized clinical trial with a control and an active treatment. We focused on effect modification by a binary covariate, using smoking as an example throughout the paper. We simulated different baseline risk levels for non-smokers and smokers; for example, mortality rates may be different. We assumed absence of treatment effect in non-smokers and varied the magnitude of the treatment effect in smokers (absent, medium, or large), reflecting the subgroup effect. We also varied the magnitude of the additional between-study heterogeneity in treatment effect (absent, medium, or large) and the magnitude of ecological bias (none: 0, quantitative: + 100% of the subgroup effect (favouring treatment effect), qualitative: − 200% of the subgroup effect (favouring placebo effect)) in the scenarios with large and medium subgroup effects. Since the subgroup effect in the no-subgroup effect scenarios is by definition equal to 0, we used the medium subgroup-effect settings to define the size of ecological bias. We also varied the number of trials [5 or 10], and the number of participants (50, 100, or 200 per trial). In total, this resulted in 486 scenarios. Per scenario we generated 1000 IPD-sets, with equal treatment allocation in each trial. We varied the percentage of smokers over the trials in order to reflect variability in the prevalence of the potential effect modifier across datasets. Specifically, the percentages were 30, 40, 50, 60, and 70%. We generated the individual outcomes using a generalized linear model (GLM) with a normal distribution with a standard deviation of 1 and an identity link for the continuous outcomes, a Bernoulli distribution and logit link for the binary outcomes, and an exponential distribution and log link for the time-to-event outcomes. We assumed a common baseline effect across studies for the intercept term (b_0_), a common prognostic effect of smoking (b_S_), and the interaction between treatment status and smoking (b_x_). The coefficients we used varied per type of outcome and scenario, see Table 1. For the treatment effect (b_T_), we generated random effects across studies (H_j_). The linear predictor in the GLM was:

**Table 1:** Parameters used in data generation

Parameters						
Type of outcome	*b*_*0*_	*b*_*T*_	*P*_*S*_	*b*_*x*_ (Large subgroup effect)	*b*_*x*_ (Medium subgroup effect)	*b*_*x*_ (No subgroup effect)
Continuous	0	0	1	−0.5	− 0.25	0
Binary (log-scale)	- 1.385	0	0.98	- 0.98	- 0.44	0
Time-to-event (log-scale)	−6.2	0	0.7	−0.7	−0.35	0

1 $$ {\displaystyle \begin{array}{l}{\mathbf{linpred}}_{\boldsymbol{ij}}={\mathbf{b}}_{\mathbf{0}}+\left({\mathbf{b}}_{\boldsymbol{T}}+{\mathbf{H}}_{\boldsymbol{j}}\right)\times {\mathbf{Treatment}}_{\boldsymbol{ij}}+{\mathbf{b}}_{\boldsymbol{S}}\times {\mathbf{Smoking}}_{\mathbf{ij}}+{\mathbf{b}}_{\mathbf{A}}\times {\mathbf{Treatment}}_{\boldsymbol{ij}}\times \overline{{\mathbf{Smoking}}_{\boldsymbol{j}.}}\\ {}{\mathbf{b}}_{\boldsymbol{w}}\times {\mathbf{Treatment}}_{\boldsymbol{ij}}\times \left({\mathbf{Smoking}}_{\boldsymbol{ij}}\hbox{-} {\mathbf{Smoking}}_{\boldsymbol{j}}\right)\end{array}} $$

where *i* denotes the participant and *j* the study. H_j_ was drawn from a normal distribution with a mean of 0 and a standard deviation (τ) of 0 (no heterogeneity), 0.25 (medium) or 0.5 (large heterogeneity), reflecting values of τ in the Cochrane Database of Systematic Reviews of 2009–2013 [19]. Note that H_j_ reflects additional between-study heterogeneity, on top of variability due to within-study sampling (imprecision) or subgroup effects.

For the continuous outcomes, the average outcome in the control group was 0 and 1 for non-smokers and smokers respectively. For the binary outcomes, in the control group the event rates of the non-smokers and smokers were respectively 20 and 40%. In logit-scale the above-mentioned event rates were approximately - 1.385 and − 0.4 respectively (see Table 1). For the time-to-event outcome, the hazard rates in the control group were defined as 2 and 4 events per 1000 person-days for non-smokers and smokers, respectively. Therefore, the increase in the hazard risk of the smokers was 0.7 on the log scale (see Table 1). For all types of outcomes, the treatment reduced the average outcome only in the smoker’s group by 0% for the no subgroup effect scenario, 25% for the medium subgroup effect scenario, and 50% for the large subgroup effect scenario. For the continuous outcome this resulted in average values of 1, 0.75, and 0.5, for the binary outcome in event rates of 40, 30, and 20%, and for the time-to-event outcome in 4, 3, and 2 events per 1000 person-days in smokers, for the no, medium, and large subgroup effect, respectively.

### Statistical approaches

Each of the 486 distinct scenarios was generated 1000 times. All 486,000 simulated data-sets were analysed using aforementioned five approaches (more details below). All analyses were conducted with the statistical package R, version 3.4.1 [20] using for one-stage approaches lmer [21], glmer [21] and coxme [22] while for two-stage approaches the packages metafor [23], logistf [24], coxphf [25].

#### Centred IPD-MA

One-stage approaches jointly analyse the IPD from all trials, accounting for the clustering of participants within trials. In line with recent recommendations [5], effect modifiers should be centred by their mean value in each trial, in order to separate the within and across-trial information. In our simulations, the percentage of smokers **p**_**j**_ is used to adjust for potential between-trial differences. Therefore, we fitted a mixed effects model as in Section 2.2.4, but now with two interaction terms to separate across- and within-trial information, thus accounting for potential ecological bias.

The statistical model is the following:

2 $$ \boldsymbol{g}\left({\boldsymbol{Y}}_{\boldsymbol{ij}}\right)={\boldsymbol{\beta}}_{\mathbf{0}\boldsymbol{j}}+{\boldsymbol{\beta}}_{\mathbf{1}\boldsymbol{j}}\times \boldsymbol{Treatmen}{\boldsymbol{t}}_{\boldsymbol{ij}}+{\boldsymbol{\beta}}_{\mathbf{2}}\times \boldsymbol{Smokin}{\boldsymbol{g}}_{\boldsymbol{ij}}+{\boldsymbol{\beta}}_{\boldsymbol{A}}\times {\boldsymbol{p}}_{\boldsymbol{j}}\times \boldsymbol{Treatmen}{\boldsymbol{t}}_{\boldsymbol{ij}}+{\boldsymbol{\beta}}_{\boldsymbol{w}}\times \boldsymbol{Treatmen}{\boldsymbol{t}}_{\boldsymbol{ij}}\times \left(\boldsymbol{Smokin}{\boldsymbol{g}}_{\boldsymbol{ij}}-{\boldsymbol{p}}_{\boldsymbol{j}}\ \right) $$

$$ {\boldsymbol{\beta}}_{\mathbf{0}\boldsymbol{j}}\sim \boldsymbol{N} \left({\boldsymbol{\beta}}_{\mathbf{0}},{\boldsymbol{\tau}}_{{\mathbf{0}}^{\mathbf{2}}}\right) $$

$$ {\boldsymbol{\beta}}_{\mathbf{1}\boldsymbol{j}}\sim \boldsymbol{N} \left({\boldsymbol{\beta}}_{\mathbf{1}},{\boldsymbol{\tau}}_{{\mathbf{1}}^{\mathbf{2}}}\right) $$

where β_A_ is the across studies interaction and β_W_ is the within-study interaction effect.

We assumed a common (fixed) effect for β_w_, as we have not generated between-study heterogeneity on the treatment-smoking interaction term. We extracted $\hat{\beta}$_w,_ which gives the interaction effect (free of ecological bias) and its corresponding *p*-value for power, estimate bias and false positive rate (FPR) calculations.

#### Naive IPD-MA

For continuous outcomes we applied linear mixed-effect models, for binary outcomes logistic mixed-effect models and for time-to-event outcomes CoxPH mixed effects models. We used all available data in a single model containing a random effect for treatment and fixed effects for intercept, subgroup and treatment-subgroup interaction. The statistical models are based on the following specification:

3 $$ \boldsymbol{g}\left({\boldsymbol{Y}}_{\boldsymbol{ij}}\right)={\boldsymbol{\beta}}_{\mathbf{0}\boldsymbol{j}}+{\boldsymbol{\beta}}_{\mathbf{1}\boldsymbol{j}}\times \boldsymbol{Treatmen}{\boldsymbol{t}}_{\boldsymbol{ij}}+{\boldsymbol{\beta}}_{\mathbf{2}}\times \boldsymbol{Smokin}{\boldsymbol{g}}_{\boldsymbol{ij}}+{\boldsymbol{\beta}}_{\boldsymbol{x}}\times \boldsymbol{Treatmen}{\boldsymbol{t}}_{\boldsymbol{ij}}\times \boldsymbol{Smokin}{\boldsymbol{g}}_{\boldsymbol{ij}} $$

$$ {\boldsymbol{\beta}}_{\mathbf{0}\boldsymbol{j}}\sim \boldsymbol{N} \left({\boldsymbol{\beta}}_{\mathbf{0}},{\boldsymbol{\tau}}_{{\mathbf{0}}^{\mathbf{2}}}\right) $$

$$ {\boldsymbol{\beta}}_{\mathbf{1}\boldsymbol{j}}\sim \boldsymbol{N} \left({\boldsymbol{\beta}}_{\mathbf{1}},{\boldsymbol{\tau}}_{{\mathbf{1}}^{\mathbf{2}}}\right) $$

We assumed common effects for β_2_ and β_x_ and random effects for β_0j_ and β_1j_. We extracted the $\hat{\beta}$_x_ estimate, which reflects the treatment-smoking interaction, and its corresponding *p*-value for power, estimate bias and false positive rate (FPR) calculations. This one-stage approach is characterised as naive [6, 16], as it does not account for potential ecological bias that may come of unadjusted confounders on trial level, like potential age-differences between trials.

#### Per-subgroup meta-analysis (PS-MA)

We separated the IPD-set for each trial into smoking and non-smoking participants. Per trial, we fitted an appropriate model per outcome: linear, logistic or CoxPH regression. In order to account for single monotone likelihood issues due to zero cells in binary and time to event outcomes, we adopted the Firth’s bias correction for logistic [20, 24] and CoxPH [21, 25] regression. This penalization method reduces the bias that occurs when adopting maximum likelihood estimation (MLE) in finite samples, and has therefore been recommended in (relatively) small datasets [26].

The mathematical description of our GLM model per trial *j* is:

4 $$ \boldsymbol{g}{\left({\boldsymbol{Y}}_{\boldsymbol{ij}}\right)}_{\boldsymbol{Nonsmokers}}={\boldsymbol{\alpha}}_{\boldsymbol{j}}+{\boldsymbol{\beta}}_{\boldsymbol{j}}\times \boldsymbol{Treatmen}{\boldsymbol{t}}_{\boldsymbol{ij}} $$

5 $$ \boldsymbol{g}{\left({\boldsymbol{Y}}_{\boldsymbol{ij}}\right)}_{\boldsymbol{Smokers}}={\boldsymbol{\gamma}}_{\boldsymbol{j}}+{\boldsymbol{\delta}}_{\boldsymbol{j}}\times \boldsymbol{Treatmen}{\boldsymbol{t}}_{\boldsymbol{ij}} $$

In the formula above, g represents the appropriate link function (identity, logit or log in case of continuous, binary or count outcomes, respectively).

Per subgroup we applied a random-effects meta-analysis with the extracted treatment coefficients using the EB method, which is equivalent to the Paule-Mandel method [27], for the τ^2^ estimation [28] and the HKSJ adjustment [29, 30]. The resulting per-subgroup pooled estimates were compared with each other with a Wald test. We extracted the differences between the per-subgroup pooled estimates and its corresponding *p*-value for power, estimate bias and false positive rate (FPR) calculations

#### Meta-analysis of interaction terms (MA-IT)

In meta-analysis of interaction terms (MA-IT), the interaction between the potential effect modifier (here smoking status) and treatment is directly modelled per trial. We hereto fitted an appropriate model per trial: linear, logistic or CoxPH regression, including a treatment-smoking interaction term. Again, we applied Firth’s bias correction for logistic [24, 31] and CoxPH [25, 32] regression.

The statistical model per trial *j* is as follows:

6 $$ \boldsymbol{g}\left({\boldsymbol{Y}}_{\boldsymbol{ij}}\right)={\boldsymbol{\beta}}_{\mathbf{0}\boldsymbol{j}}+{\boldsymbol{\beta}}_{\boldsymbol{Tj}}\times \boldsymbol{Treatmen}{\boldsymbol{t}}_{\boldsymbol{ij}}+{\boldsymbol{\beta}}_{\boldsymbol{sj}}\times \boldsymbol{Smokin}{\boldsymbol{g}}_{\boldsymbol{ij}}+{\boldsymbol{\beta}}_{\boldsymbol{xj}}\times \boldsymbol{Treatmen}{\boldsymbol{t}}_{\boldsymbol{ij}}\times \boldsymbol{Smokin}{\boldsymbol{g}}_{\boldsymbol{ij}} $$

We applied a fixed effect meta-analysis for pooling the $\hat{\beta}$_xj_ estimates of interaction. We extracted the pooled estimate and its corresponding *p*-value for power, estimate bias and false positive rate (FPR) calculations.

#### Meta-regression

Meta-regression is a two-stage approach that uses weighted regression to associate the effect of a trial-level moderator variable (i.e. the percentage of smokers per trial) with the estimated treatment effect in that trial. Hereto, each trial is first analysed separately using either linear, logistic or Cox proportional hazards (CoxPH) regression. Again, we applied Firth’s bias correction for logistic [24, 31] and CoxPH [25, 32] regression. Subsequently, for each trial the estimated treatment effect $\hat{\beta}$_j_ and the percentage of smokers p_j_ are extracted. We evaluated the presence of subgroups by fitting a linear mixed model with as dependent variable the extracted treatment coefficients $\hat{\beta}$_j_ and as explanatory variable the percentage of smokers p_j_. Between-study variation in treatment effects was modelled with a random intercept. Weights were based on the inverse variances of $\hat{\beta}$_j_. We applied the empirical Bayes (EB) (Paule-Mandel) method for the estimation of the between-study heterogeneity τ^2^ and performed the Hartung-Knapp-Sidik-Jonkman (HKSJ) adjustment [28, 29].

The mathematical form of our model is:

7 $$ {\displaystyle \begin{array}{c}\hat{\beta}\mathbf{j}={\boldsymbol{\gamma}}_{0\boldsymbol{j}}+{\boldsymbol{\gamma}}_1\times {\boldsymbol{p}}_{\boldsymbol{j}}+{\boldsymbol{\varepsilon}}_{\boldsymbol{j}}\\ {}{\boldsymbol{\varepsilon}}_{\boldsymbol{j}}\sim \boldsymbol{N}\left(0,{\boldsymbol{\sigma}}^2\ \right)\\ {}{\boldsymbol{\gamma}}_{0\boldsymbol{j}}\sim \boldsymbol{N}\left({\boldsymbol{\gamma}}_0,{\boldsymbol{\tau}}_{0^2}\ \right)\end{array}} $$

where ***γ***_**0*j***_***,γ***_**1**_ are the meta-regression coefficients and ε_j_ the residual error of study *j*. We extracted ${\hat{\gamma}}_1$ and its corresponding *p*-value for power, estimate bias and false positive rate (FPR) calculations, see section 2.3.

### Methods comparison

**Fig. 1:**
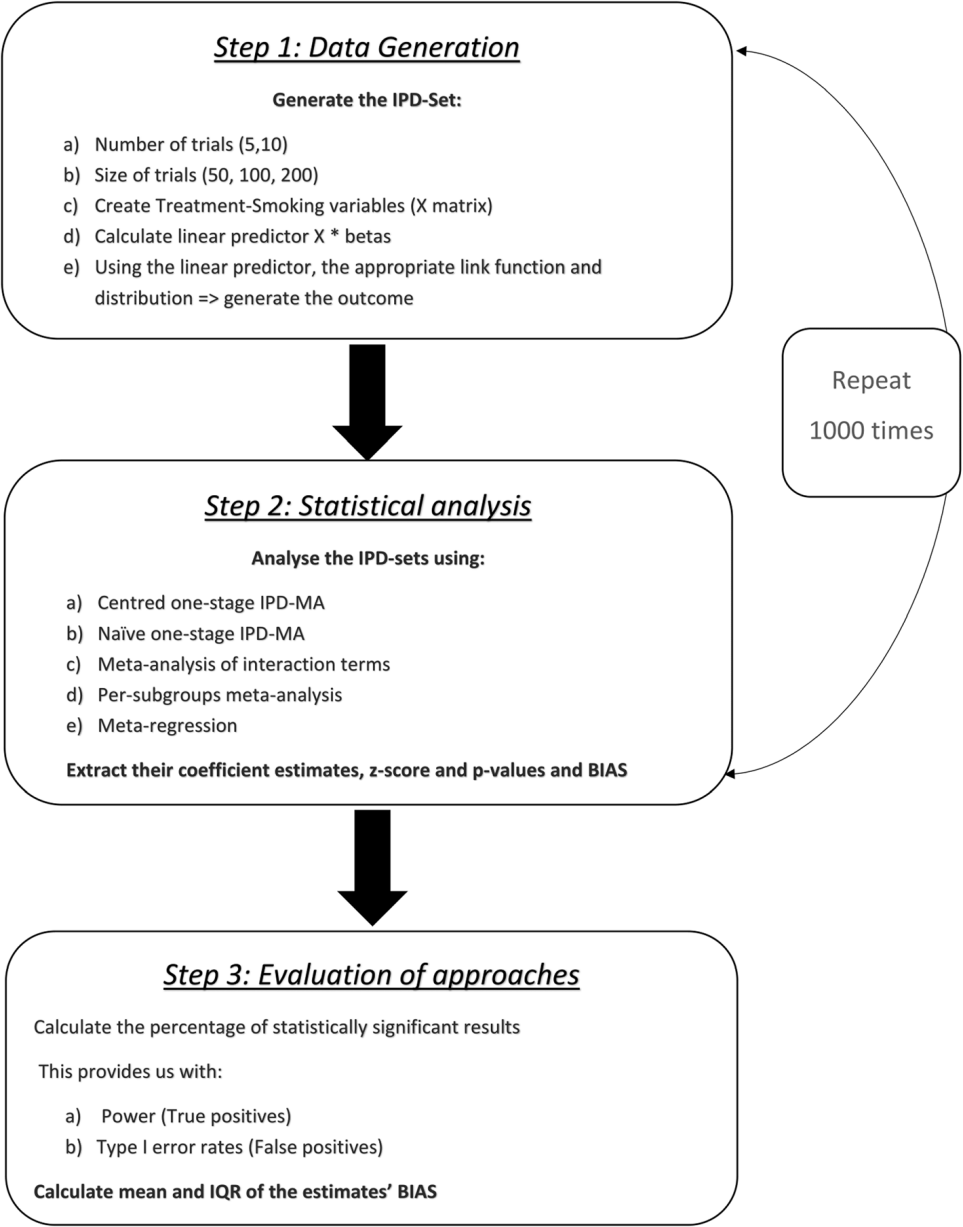
Overview of simulations approximately here

To assess the power, FPRs and bias in the subgroup effect estimates of all approaches, each scenario was repeated 1000 times, and we analysed the data as described in sections 2.2.1–2.2.5. See also Fig. 1, which summarises our simulation procedure.

The power of a statistical test is the probability that the test correctly rejects the null hypothesis (H_0_). As we are comparing the approaches in a simulation setting, we know the direction of the subgroup effect, if any. Therefore, we conducted a one-sided test with a significance level of 0.025 in the scenarios with the medium or large subgroup effects to assess the power of the approaches. As we applied Firth’s bias correction, all approaches converged, and we defined power as the percentage of significant results, based on all simulations.

The FPR is the probability of finding a statically significant subgroup effect where there is none. Therefore, we conducted a two-sided hypothesis test with a nominal significance level of 0.05 in all scenarios without subgroup effect and calculated the percentage of statistically significant results.

The estimand we are investigating is the treatment-effect modification term. In our data-generation mechanism that would be the equivalent to the interaction term **b**_***w***_ see formula [1]. Each of our aforementioned approaches estimates this treatment effect modification term in a different manner. Per approach, we calculated the bias in the estimate of the resulting coefficient, a difference between the estimand and the coefficient estimate (${\mathbf{b}}_{\boldsymbol{w}}-\hat{{\boldsymbol{\beta}}_{\boldsymbol{method}}}$).

## Results

**Fig. 2:**
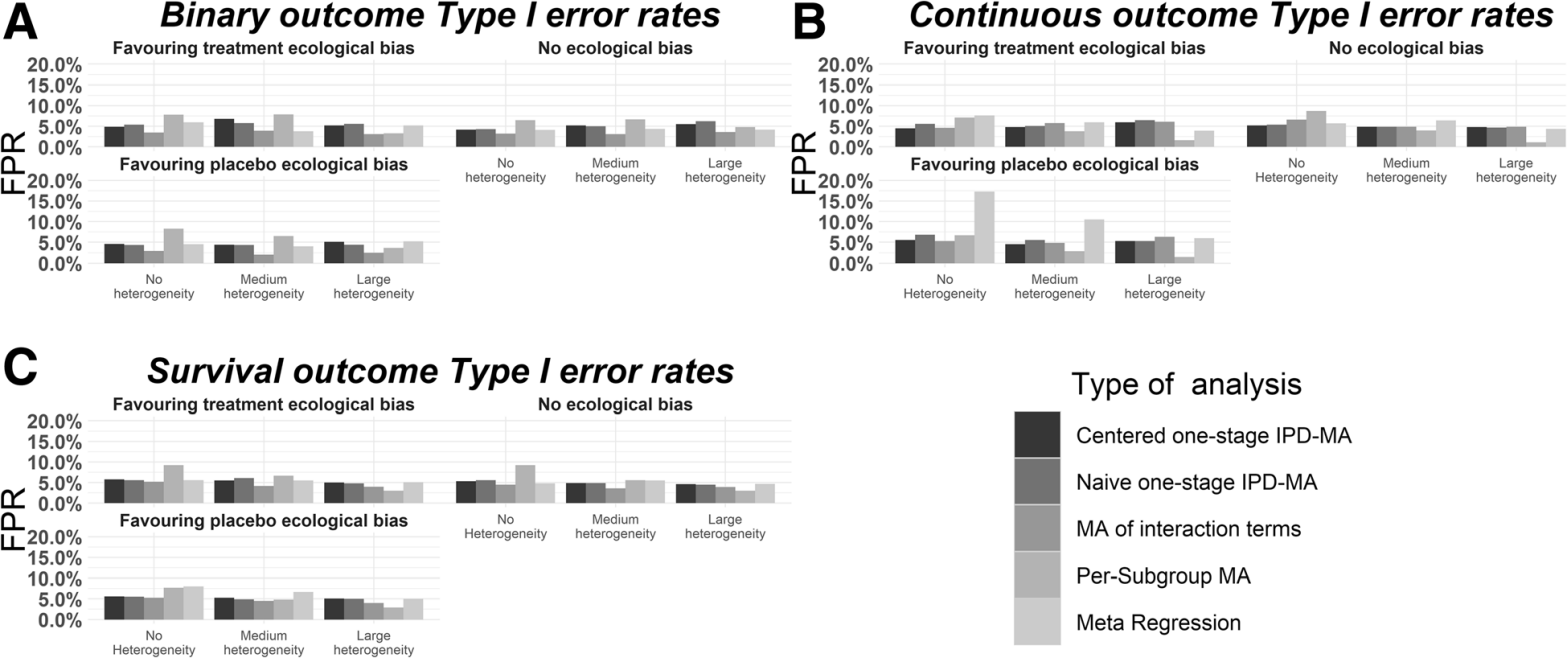
Type I errors for the scenarios of 5 studies with 100 participants each approximately here

**Fig. 3:**
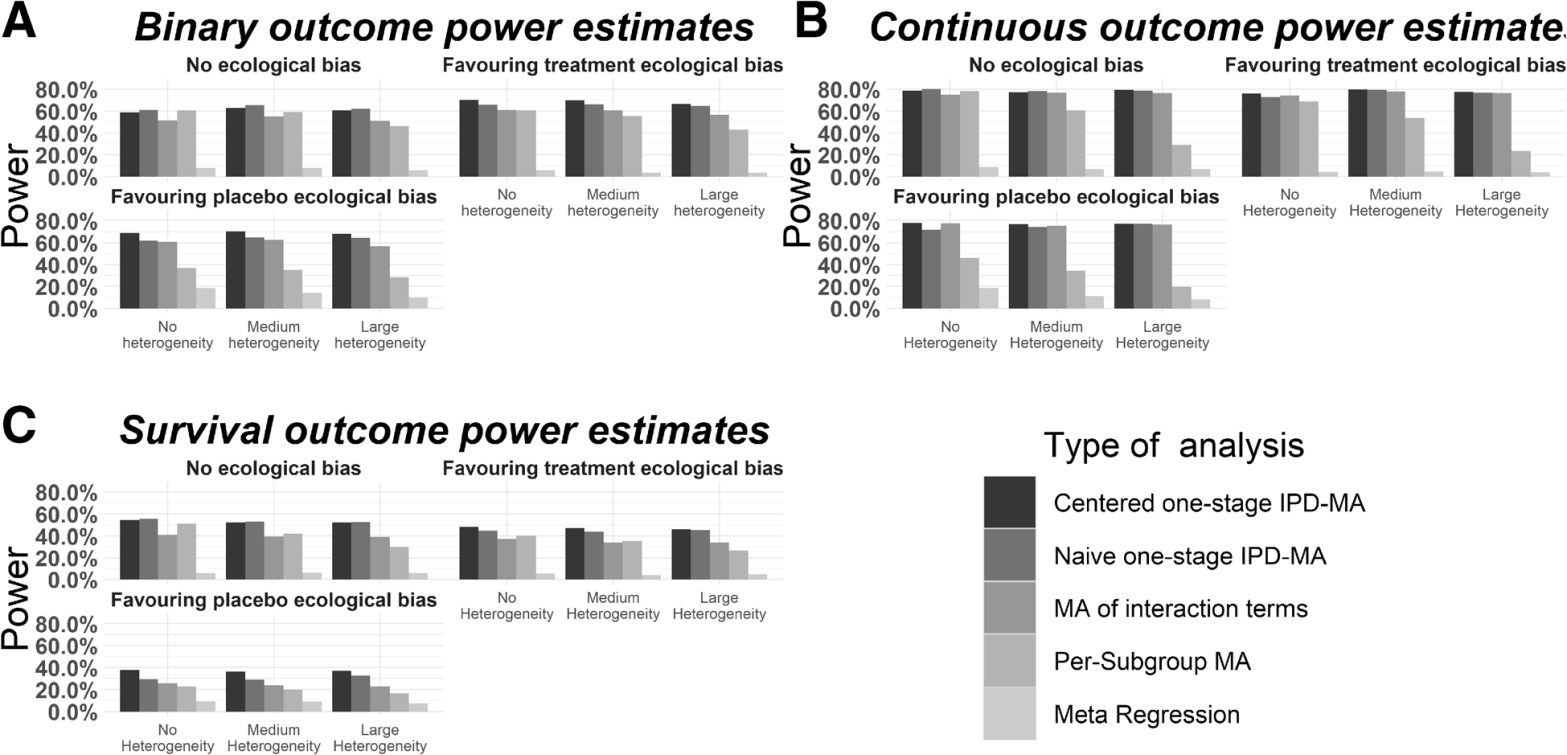
Power to detect a large subgroup effect in the scenarios of 5 studies with 100 participants each approximately here

For illustrative purposes we show the power and FPR results of our simulations for the scenarios of five trials with each 100 participants in Figs. 2 and 3. Furthermore, we show the bias for each method into Table 2, Table 3, Table 4, Table 5, and Table 6. The above setting was considered most representative for typical IPD-MA. The results of other scenarios are shown in the appendices (Additional files 1, 2, 3, 4, 5, 6, 7, 8 and 9). Results were similar to Figs. 2 and 3, but with an increasing trend in power as subgroup effect, number of participants or number of trials increased.

**Table 2:** Centred CPD-MA coefficient estimate bias (mean [IQR])

		Centred one-stage IPD-MA BIAS [IQR]								
		Binary outcome			Continuous outcome			Survival outcome		
Heterogeneity magnitude	Ecological Bias	Large subgroup effect	Medium subgroup effect	No sugroup effect	Large subgroup effect	Medium subgroup effect	No sugroup effect	Large subgroup effect	Medium subgroup effect	No sugroup effect
No heterogeneity	No ecological bias	-0.008 [0.592]	-0.004 [0.626]	0.007 [0.533]	-0.006 [0.257]	-0.012 [0.254]	0 [0.24]	0.024 [0.459]	0.017 [0.47]	-0.012 [0.393]
No heterogeneity	Favouring treatment ecological bias	-0.038 [0.572]	-0.001 [0.596]	0.002 [0.578]	0.005 [0.259]	0.006 [0.253]	-0.001 [0.238]	0.019 [0.505]	0.019 [0.486]	-0.008 [0.423]
No heterogeneity	Favouring placebo ecological bias	-0.013 [0.566]	0.002 [0.567]	-0.013 [0.589]	0.001 [0.245]	0.005 [0.238]	-0.004 [0.245]	0.023 [0.599]	0.021 [0.539]	-0.013 [0.371]
Medium heterogeneity	No ecological bias	-0.042 [0.618]	0.024 [0.533]	0.008 [0.598]	-0.001 [0.251]	-0.009 [0.237]	-0.005 [0.234]	0 [0.453]	-0.002 [0.466]	-0.012 [0.368]
Medium heterogeneity	Favouring treatment ecological bias	-0.048 [0.563]	-0.013 [0.574]	0.01 [0.599]	-0.01 [0.242]	0.006 [0.258]	0.004 [0.256]	0.002 [0.485]	-0.003 [0.484]	-0.013 [0.4]
Medium heterogeneity	Favouring placebo ecological bias	-0.024 [0.553]	-0.038 [0.525]	-0.02 [0.56]	0.003 [0.246]	0 [0.246]	-0.01 [0.266]	-0.012 [0.62]	-0.017 [0.545]	-0.011 [0.382]
Large heterogeneity	No ecological bias	-0.007 [0.611]	-0.025 [0.545]	0.006 [0.607]	-0.002 [0.251]	0.003 [0.263]	0.001 [0.243]	0.006 [0.467]	-0.001 [0.474]	-0.011 [0.388]
Large heterogeneity	Favouring treatment ecological bias	-0.02 [0.554]	-0.008 [0.568]	-0.001 [0.624]	0.001 [0.241]	-0.008 [0.251]	0.006 [0.244]	0 [0.492]	-0.007 [0.48]	-0.011 [0.394]
Large heterogeneity	Favouring placebo ecological bias	-0.01 [0.577]	-0.02 [0.553]	-0.006 [0.587]	0.004 [0.248]	-0.002 [0.249]	-0.002 [0.225]	-0.011 [0.592]	-0.01 [0.557]	-0.012 [0.366]

**Table 3:** Naïve CPD-MA coefficient estimate bias (mean [IQR])

		Naive one-stage IPD-MA BIAS [IQR]								
		Binary outcome			Continuous outcome			Survival outcome		
Heterogeneity magnitude	Ecological Bias	Large subgroup effect	Medium subgroup effect	No sugroup effect	Large subgroup effect	Medium subgroup effect	No sugroup effect	Large subgroup effect	Medium subgroup effect	No sugroup effect
No heterogeneity	No ecological bias	-0.012 [0.595]	-0.001 [0.596]	0 [0.516]	-0.005 [0.258]	-0.014 [0.25]	0 [0.231]	0.017 [0.459]	0.011 [0.456]	-0.013 [0.377]
No heterogeneity	Favouring treatment ecological bias	0.021 [0.56]	0.027 [0.57]	-0.006 [0.596]	0.031 [0.255]	0.019 [0.24]	-0.027 [0.242]	-0.03 [0.489]	-0.009 [0.474]	-0.03 [0.409]
No heterogeneity	Favouring placebo ecological bias	0.075 [0.596]	0.073 [0.566]	-0.013 [0.585]	0.042 [0.241]	0.041 [0.232]	0.035 [0.247]	-0.089 [0.572]	-0.047 [0.51]	0.027 [0.367]
Medium heterogeneity	No ecological bias	-0.043 [0.627]	0.023 [0.523]	0.004 [0.562]	-0.001 [0.247]	-0.01 [0.239]	-0.007 [0.228]	-0.002 [0.449]	-0.002 [0.464]	-0.014 [0.365]
Medium heterogeneity	Favouring treatment ecological bias	-0.003 [0.544]	0.009 [0.565]	0.007 [0.599]	0 [0.249]	0.011 [0.257]	-0.005 [0.257]	-0.031 [0.491]	-0.021 [0.479]	-0.029 [0.398]
Medium heterogeneity	Favouring placebo ecological bias	0.052 [0.572]	0.013 [0.516]	-0.024 [0.563]	0.024 [0.255]	0.015 [0.245]	0.008 [0.262]	-0.107 [0.589]	-0.068 [0.523]	0.009 [0.383]
Large heterogeneity	No ecological bias	-0.01 [0.59]	-0.027 [0.551]	0.007 [0.591]	-0.003 [0.255]	0.002 [0.268]	0.002 [0.242]	0.005 [0.456]	-0.002 [0.463]	-0.012 [0.389]
Large heterogeneity	Favouring treatment ecological bias	0.009 [0.559]	0.003 [0.557]	-0.004 [0.586]	0.005 [0.241]	-0.006 [0.255]	0.003 [0.249]	-0.019 [0.489]	-0.016 [0.483]	-0.018 [0.392]
Large heterogeneity	Favouring placebo ecological bias	0.044 [0.569]	0.015 [0.56]	-0.008 [0.591]	0.012 [0.242]	0.001 [0.247]	0.004 [0.23]	-0.071 [0.565]	-0.040 [0.542]	-0.004 [0.365]

**Table 4:** Meta-analysis of interaction terms coefficient estimate bias (mean [IQR])

		MA-IT BIAS [IQR]								
		Binary outcome			Continuous outcome			Survival outcome		
Heterogeneity magnitude	Ecological Bias	Large subgroup effect	Medium subgroup effect	No sugroup effect	Large subgroup effect	Medium subgroup effect	No sugroup effect	Large subgroup effect	Medium subgroup effect	No sugroup effect
No heterogeneity	No ecological bias	0.044 [0.573]	0.015 [0.578]	0.002 [0.496]	-0.006 [0.277]	-0.011 [0.252]	0.002 [0.25]	-0.032 [0.461]	-0.014 [0.46]	-0.014 [0.408]
No heterogeneity	Favouring treatment ecological bias	0.028 [0.543]	0.03 [0.563]	-0.003 [0.568]	0.005 [0.273]	0.007 [0.253]	0 [0.241]	-0.036 [0.471]	-0.01 [0.467]	-0.018 [0.424]
No heterogeneity	Favouring placebo ecological bias	0.06 [0.54]	0.056 [0.541]	-0.019 [0.576]	0 [0.25]	0.007 [0.244]	-0.006 [0.254]	-0.038 [0.553]	0.003 [0.513]	-0.005 [0.374]
Medium heterogeneity	No ecological bias	0.018 [0.577]	0.039 [0.514]	0.008 [0.54]	-0.003 [0.26]	-0.008 [0.249]	-0.006 [0.237]	-0.058 [0.461]	-0.0390 [0.467]	-0.016 [0.386]
Medium heterogeneity	Favouring treatment ecological bias	0.017 [0.532]	0.015 [0.538]	0.007 [0.567]	-0.01 [0.259]	0.005 [0.259]	0.003 [0.25]	-0.058 [0.48]	-0.0390 [0.497]	-0.025 [0.402]
Medium heterogeneity	Favouring placebo ecological bias	0.046 [0.53]	0.006 [0.497]	-0.019 [0.533]	0.002 [0.25]	0.003 [0.26]	-0.01 [0.268]	-0.077 [0.566]	-0.0381 [0.511]	-0.005 [0.37]
Large heterogeneity	No ecological bias	0.049 [0.556]	-0.004 [0.519]	0.003 [0.573]	-0.001 [0.258]	0.002 [0.281]	0.003 [0.257]	-0.053 [0.451]	-0.039 [0.472]	-0.016 [0.38]
Large heterogeneity	Favouring treatment ecological bias	0.048 [0.538]	0.016 [0.525]	-0.008 [0.588]	-0.001 [0.255]	-0.008 [0.271]	0.006 [0.256]	-0.063 [0.497]	-0.0431 [0.492]	-0.024 [0.403]
Large heterogeneity	Favouring placebo ecological bias	0.063 [0.563]	0.023 [0.508]	-0.012 [0.56]	0.004 [0.25]	-0.003 [0.257]	-0.003 [0.248]	-0.077 [0.541]	-0.0350 [0.532]	-0.006 [0.376]

**Table 5:** Per-subgroup meta-analysis coefficient estimate bias (mean [IQR])

		PS-MA BIAS [IQR]								
		Binary outcome			Continuous outcome			Survival outcome		
Heterogeneity magnitude	Ecological Bias	Large subgroup effect	Medium subgroup effect	No sugroup effect	Large subgroup effect	Medium subgroup effect	No sugroup effect	Large subgroup effect	Medium subgroup effect	No sugroup effect
No heterogeneity	No ecological bias	0.053 [0.526]	0.02 [0.542]	-0.002 [0.471]	-0.005 [0.263]	-0.014 [0.255]	0.001 [0.245]	-0.058 [0.437]	-0.032 [0.42]	-0.011 [0.404]
No heterogeneity	Favouring treatment ecological bias	0.102 [0.516]	0.063 [0.522]	-0.006 [0.529]	0.034 [0.264]	0.02 [0.238]	-0.028 [0.242]	-0.112 [0.426]	-0.050 [0.438]	-0.04 [0.417]
No heterogeneity	Favouring placebo ecological bias	0.216 [0.515]	0.132 [0.516]	-0.013 [0.537]	0.071 [0.24]	0.048 [0.25]	0.047 [0.25]	-0.21 [0.511]	-0.090 [0.46]	0.045 [0.364]
Medium heterogeneity	No ecological bias	0.03 [0.554]	0.038 [0.486]	-0.002 [0.525]	-0.003 [0.247]	-0.009 [0.24]	-0.009 [0.238]	-0.072 [0.438]	-0.041 [0.462]	-0.021 [0.38]
Medium heterogeneity	Favouring treatment ecological bias	0.085 [0.511]	0.043 [0.523]	-0.001 [0.544]	0.007 [0.247]	0.016 [0.253]	-0.014 [0.254]	-0.119 [0.453]	-0.066 [0.455]	-0.048 [0.401]
Medium heterogeneity	Favouring placebo ecological bias	0.188 [0.532]	0.078 [0.482]	-0.03 [0.53]	0.055 [0.247]	0.031 [0.26]	0.027 [0.267]	-0.228 [0.533]	-0.105 [0.481]	0.03 [0.364]
Large heterogeneity	No ecological bias	0.049 [0.525]	-0.019 [0.515]	-0.02 [0.55]	-0.003 [0.268]	0.002 [0.276]	0.005 [0.247]	-0.064 [0.464]	-0.031 [0.473]	-0.032 [0.395]
Large heterogeneity	Favouring treatment ecological bias	0.099 [0.518]	0.028 [0.515]	-0.03 [0.548]	0.011 [0.261]	-0.003 [0.266]	-0.003 [0.236]	-0.112 [0.467]	-0.051 [0.465]	-0.056 [0.408]
Large heterogeneity	Favouring placebo ecological bias	0.176 [0.515]	0.076 [0.53]	-0.031 [0.534]	0.029 [0.265]	0.009 [0.264]	0.019 [0.245]	-0.207 [0.549]	-0.082 [0.493]	0.005 [0.371]

**Table 6:** Meta-regression coefficient estimate bias (mean [IQR])

		Meta regression BIAS [IQR]								
		Binary outcome			Continuous outcome			Survival outcome		
Heterogeneity magnitude	Ecological Bias	Large subgroup effect	Medium subgroup effect	No sugroup effect	Large subgroup effect	Medium subgroup effect	No sugroup effect	Large subgroup effect	Medium subgroup effect	No sugroup effect
No heterogeneity	No ecological bias	-0.026 [2.083]	0.029 [1.973]	-0.06 [1.893]	-0.009 [0.958]	-0.052 [0.935]	-0.021 [0.911]	-0.085 [1.549]	-0.081 [1.644]	0.027 [1.366]
No heterogeneity	Favouring treatment ecological bias	1.022 [1.872]	0.5 [1.835]	-0.084 [1.784]	0.516 [0.912]	0.236 [0.892]	-0.509 [0.97]	-0.748 [1.71]	-0.399 [1.657]	-0.312 [1.356]
No heterogeneity	Favouring placebo ecological bias	2.984 [1.873]	1.276 [1.73]	0.076 [1.8]	1.515 [0.921]	0.742 [0.94]	1.006 [0.93]	-2.045 [1.95]	-1.058 [1.689]	0.701 [1.333]
Medium heterogeneity	No ecological bias	0.01 [2.234]	0.051 [2.067]	-0.046 [1.976]	0 [1.411]	-0.008 [1.483]	-0.026 [1.365]	-0.013 [1.945]	0.007 [1.984]	-0.03 [1.749]
Medium heterogeneity	Favouring treatment ecological bias	1.008 [2.157]	0.468 [2.037]	0.019 [2.104]	0.449 [1.339]	0.235 [1.467]	-0.468 [1.437]	-0.689 [2.037]	-0.342 [2.019]	-0.363 [1.738]
Medium heterogeneity	Favouring placebo ecological bias	2.963 [2.133]	1.296 [2.093]	-0.069 [2.014]	1.525 [1.373]	0.724 [1.43]	0.998 [1.479]	-2.011 [2.185]	-1.002 [2.099]	0.633 [1.72]
Large heterogeneity	No ecological bias	-0.067 [2.839]	0.024 [2.92]	0.021 [2.55]	-0.063 [2.44]	-0.046 [2.295]	0.101 [2.37]	0.007 [2.614]	0.023 [2.682]	-0.056 [2.577]
Large heterogeneity	Favouring treatment ecological bias	1.014 [2.737]	0.516 [2.764]	0.019 [2.81]	0.536 [2.179]	0.195 [2.22]	-0.404 [2.252]	-0.669 [2.633]	-0.298 [2.678]	-0.39 [2.521]
Large heterogeneity	Favouring placebo ecological bias	2.883 [2.701]	1.34 [2.723]	0.038 [2.721]	1.426 [2.454]	0.656 [2.562]	1.033 [2.264]	-1.984 [2.784]	-0.984 [2.719]	0.617 [2.491]

### False positive rates

Figure 2 shows that centred and naive IPD-MA result in consistent type I error rates (around nominal 5%) for all types of outcome. However, for MA-IT we noticed that FPRs were low (around 2.5 to 3.5%) when modelling binary outcomes. PS-MA yielded high FPRs in scenarios without heterogeneity, reaching approximately 9% for survival outcomes. But when heterogeneity increased, PS-MA’s FPRs decreased even below 5%, reaching 1% for continuous outcomes. Finally, meta-regression showed type I error rates approximately at 5% for all types of outcome.

For scenarios with ecological bias, centred and naïve IPD-MA showed slightly reduced FPRs for the binary outcomes in scenarios without heterogeneity. In the other scenarios they performed as described. MA-IT results were not affected by the addition of ecological bias. PS-MA showed mixed results. For binary outcomes the addition of ecological bias resulted in increased FPRs, while for the continuous outcomes the FPRs decreased, and for time-to-event outcomes FPRs remained unaffected. Furthermore, similar to settings without ecological bias, PS-MA showed a decreasing trend in the FPRs estimates when heterogeneity increased. Finally, meta-regression showed increased FPRs especially for continuous outcomes, reaching 18% in the scenarios favouring placebo (qualitative ecological bias).

### Power

In general, naïve and centred IPD-MA showed approximately similar results. In scenarios without ecological bias naïve IPD-MA showed slightly more power than centred IPD-MA. In scenarios with ecological bias centred IPD-MA showed more power than naïve, which increased even more in the qualitative compared to the quantitative ecological bias scenarios. Furthermore, the power of centred IPD-MA was highest in scenarios with ecological bias. Compared to IPD-MA methods MA-IT showed decreased levels of statistically significant results. In scenarios with a limited number of trials (*n* = 5) and small sample sizes (*n* = 50 participants), we observed the strongest difference. A similar increase in the power of MA-IT as seen for the centred IPD-MA was observed with the addition of ecological bias. With PS-MA, power decreased as heterogeneity increased. For continuous outcomes we observed the strongest decrease: the power dropped from 80% in scenarios without heterogeneity to 25% in highly heterogeneous scenarios. PS-MA showed decreased power in scenarios with ecological bias, compared to those without. Finally, meta-regression demonstrated low power in all scenarios and often mis-identified the direction of the interaction effect (i.e. statistically significant results for negative rather than positive interaction coefficients), especially in scenarios with qualitative ecological bias.

#### Binary outcomes

Figure 3a shows the results of our simulations for binary outcomes. In scenarios with 5 trials and 100 participants, all five statistical approaches showed less than 70% power to detect large subgroup effects. In scenarios without ecological bias centred and naive IPD-MA approaches had similar power results: around 60% power across all three heterogeneity levels. When ecological bias was added, centred IPD-MA showed an increase from 60 to 70% in power. The increase in power was less for naïve IPD-MA, from 60 to 65%. MA-IT showed lower power than the centred and naïve IPD-MA (around 50%), and remained unaffected by heterogeneity. The presence of ecological bias increased the power of MA-IT to 60%. PS-MA showed a notable decrease in power as heterogeneity increased. In scenarios without ecological bias the power dropped from 60% (no heterogeneity) to 40% (large heterogeneity). The addition of qualitative bias had a stronger effect on the power of PS-MA than quantitative bias. Specifically, in scenarios without heterogeneity, power dropped from 60 to 35% when qualitative bias was added, while power remained approximately the same with quantitative bias. Similar patterns are observed with heterogeneity. Meta-regression resulted consistently in power below 10%, except for scenarios with qualitative ecological bias, where it showed increased percentages of statistically significant results (reaching 30%), but these were mainly related to effects in the opposite direction.

#### Continuous outcomes

Figure 3b shows the results of our simulations for continuous outcomes. All five statistical approaches showed less than 80% power to detect large subgroup effects. In particular, without ecological bias centred and naïve IPD-MA approaches had similar consistent power results (approximately 80%) across all three heterogeneity levels, which slightly decreased in the scenarios with ecological bias. MA-IT showed lower power than centred and naïve IPD-MA approaches, i.e. approximately 75% and was unaffected by heterogeneity and ecological bias. PS-MA showed a decrease in power as heterogeneity increased: in scenarios without ecological bias the power dropped from nearly 80% (no heterogeneity) to around 30% (large heterogeneity). Furthermore, PS-MA showed decreased power in scenarios without heterogeneity but with quantitative and qualitative ecological bias, 70 and 45% respectively, which further dropped to 25 and 20% in scenarios with large heterogeneity. Meta-regression in scenarios with no and quantitative ecological bias resulted in power less than 10% for all heterogeneity levels. In contrast, power increased in scenarios with qualitative ecological bias, but in the opposite direction of the subgroup effect.

#### Time-to-event outcomes

Figure 3c shows the results of our simulations for time-to-event outcomes. All five statistical approaches showed less than 70% power to detect large subgroup effects. In particular, in scenarios without ecological bias centred and naïve IPD-MA approaches had similar consistent power (approximately 65%) across all three heterogeneity levels, with a slight increase in power in the scenarios with ecological bias. MA-IT showed slightly lower power than centred and naïve IPD-MA approaches, i.e. approximately 55% and this was unaffected by heterogeneity. Nevertheless, similar to the centred and naïve IPD-MA methods, MA-IT showed an increase in power in presence of ecological bias from 60 to 70%. PS-MA showed a decrease in power as heterogeneity increased: in scenarios without ecological bias the power dropped from nearly 65% (no heterogeneity) to around 45% (large heterogeneity). Furthermore, PS-MA showed decreased power in scenarios with qualitative compared to quantitative ecological bias, 35 and 60% respectively, and dropped to 30 and 45% when large heterogeneity was introduced. Meta-regression resulted in power less than 10% for all heterogeneity levels in scenarios with no or quantitative ecological bias. In contrast, meta-regression showed more power (approximately 15%) in presence of qualitative bias but in the opposite direction of the subgroup effect.

### Bias in the estimates of the treatment-effect modification

Detailed results of the bias are presented in Table 2, Table 3, Table 4, Table 5, and Table 6. The estimate bias of centred and naïve IPD-MA was nearly zero and remained unaffected by addition of ecological bias and heterogeneity in all scenarios for all outcomes. The results were approximately zero, with a maximum bias of 0.017 and minimum of − 0.005 for centred and 0.1 and − 0.07 for the naïve IPD-MA. MA-IT showed slightly increased bias for binary outcomes especially in scenarios with low numbers of participants (50) and trials [5] and a high level of heterogeneity. PS-MA amalgamated heterogeneity with ecological bias and showed bias in all scenarios and outcomes, especially on binary and survival outcomes. Finally, meta-regression estimated in all scenarios the **b**_**A**_ term, see formula 1, rather than the estimand b_w_ we were interested in. Therefore, meta-regression showed extreme bias in all scenarios with ecological bias, both quantitative and qualitative.

## Discussion

We compared five common statistical approaches for subgroup detection in IPD-MA in an extensive simulation study. Our results showed that overall the centred IPD-MA described by Hua et al. [6] performed best in terms of power, false positive rates and estimate bias, particularly in scenarios with heterogeneity and ecological bias. Both (naive and centred) one stage IPD-MA approaches reached high levels of power, with minimal bias, while retaining nominal and stable false positive rates around 5%. The MA-IT approach was less powerful, particularly in scenarios with binary outcomes, small sample sizes (*n* = 50 participants), and few trials (5 studies). PS-MA showed inconsistent power, decreasing as heterogeneity and ecological bias increased, and high FPRs in scenarios without heterogeneity. Furthermore, PS-MA showed high levels of estimate bias. Meta-regression showed a lack of power and often mis-identified the direction of the interaction effect (i.e. negative rather than positive interaction coefficients). Although our findings were based on binary, continuous and survival endpoints, it is highly likely that they will be applicable to other type of outcomes, such as counts (using different link functions such as poisson link function) and rates (using logit or arcsin transformations of the outcome or fitting a beta-regression model, which maximises the beta distribution likelihood). This supports the generalizability of our recommendations.

### Comparison with literature

Our results are in agreement with the simulation study of Lambert et al. [10], who compared in a setting without between-study heterogeneity meta-regression and naive IPD meta-analysis and showed that meta-regression is prone to a lack of power even when there is no heterogeneity in treatment effect. Our results confirm that meta-regression and PS-MA have limited usefulness to investigate subgroup effects when individual participant data are available and that centred and naïve IPD-MA approaches or MA-IT should be preferred [33]. Our findings are also in line with a previous overview study of Fisher et al., where MA-IT, PS-MA, naive and centred IPD-MA were described and fitted in empirical studies [8]. Furthermore, our results are also in agreement with empirical studies for continuous and binary outcomes [12, 14], which pointed out the need of individual participant data and stressed that different results may emerge with different approaches. In agreement with literature we have detected a lack of power in the MA-IT compared to centred and naïve IPD-MA approaches in small sample sized binary outcomes scenarios [2]. Finally, we simulated data with ecological bias using a coefficient for the across studies differences in the treatment effect (**b**_**A**_) see formula 1. This implies a linear association between the interaction of percentage of smokers and treatment with the outcome. Our results show that centred and naïve IPD-MA have minor differences in power and bias, which seems contradictory to the results of Hua et al. [6]. Nevertheless, that is due to different data generating mechanisms. We generated ecological bias proportional to the percentage of smokers, while Hua generated constant ecological bias for all studies with percentage of smokers above 50%.

### Strengths and limitations

The major strength of our simulation study is the variety of scenarios and approaches we investigated. In particular, we varied multiple elements relevant for meta-analysis, such as between-study treatment effect heterogeneity, quantitative and qualitative ecological bias, size and number of trials, and subgroup effect magnitude. To our knowledge, this is the first study comparing power, FPRs and estimate bias of centred and naive IPD-MA, MA-IT, PS-MA and meta-regression when detecting subgroup effects. The fact that we applied all approaches and scenarios on three common outcomes allows us to draw conclusions that are widely applicable. The broad set-up of the simulations allowed us also to generate similar heterogeneity, ecological bias and subgroup effects across all three types of outcome.

Some potential limitations should also be discussed. First, we only generated between-study heterogeneity for the treatment effect even though we could have generated heterogeneity on other parameters in the model. Nevertheless, intercept and smoking can be considered nuisance terms when the main interest is in investigating treatment effects, thus generating heterogeneity on the intercept and/or the smoker’s effect would not have altered our results and our conclusions. We avoided the introduction of heterogeneity in the interaction term, as that would have combined the statistically significant results resulting from our scenarios with those resulting from random noise. As a consequence, the differences in power and FPR across the methods would have been underestimated. In addition, we assumed that between-study heterogeneity was drawn from a normal distribution, such that modelling assumptions of the various methods were (partially) in agreement with the data generation mechanism. This may have led to optimistic estimates of the power of all evaluated methods. In these regards, researchers should be aware that all methods are likely to perform poorer in practice (further underlining the poor performance of meta-regression and per-subgroup analysis). Second, we assumed that trials were perfectly balanced with respect to treatment allocation. Because small trials are more likely to be imbalanced by chance, their power to detect subgroup effects and treatment-covariate interactions would be somewhat lower than is the case in our simulation study. Finally, we simulated a binary effect modifier reflecting either binary variables such as smoking or the common practice where dichotomizing continuous covariates is typically conducted. Nevertheless, effect modification is also commonly present in continuous and multilevel categorical variables, which are not covered in our simulations.

### Implications for practice

Centred and naïve IPD-MA, MA-IT and PS-MA are considered well developed and powerful statistical tools for IPD analysis. Nevertheless, we would like to highlight that these methods should be used with caution and applied by a research team involving at least one member with the appropriate statistical expertise. Centred and naïve methods need careful modelling, as some assumptions they inherently make may not be applicable to all data. Furthermore, convergence issues may emerge especially in mixed-effects models with time-to-event outcomes. MA-IT may be problematic when investigating subgroup effects across multiple trials because of lower power than the centred and naïve IPD-MA approaches. Furthermore, since in practice single trials are rarely sufficiently powered to investigate subgroups or treatment-covariate interactions, the MA-IT approach may also be hampered. For instance, practitioners may omit trials due to single monotone likelihood issues (i.e. all participants in a subgroup having the same outcome), thus reducing the power of the meta-analysis. Firth’s bias correction may fix the sparse-data bias issue but should be used with prudence as this method introduces bias in the estimates shrinking them to zero. The suboptimal performance of the PS-MA approach implies that this method should be avoided especially when between study heterogeneity and ecological bias are present, as these effects may be amalgamated within the per subgroup estimates, which may cause biased results and reduction of power.

Meta-regression is a statistical approach for aggregated data and should be avoided in IPD. It is known to have low power due to inefficient use of information especially when the subgroup covariate has a small variance across trials. Furthermore, meta-regression is severely prone to ecological bias.

MA-IT is less complicated than one-stage methods and in meta-analyses with large trials it produces similar results. Furthermore, results can be presented in terms of forest plots, in contrast to one-stage methods, where this is less obvious. Nevertheless, assuming correctly fitted models for all methods, we advocate that centred IPD-MA should be preferred. Our study demonstrates that the centred IPD-MA method yields high power and appropriate FPR, with minimal estimate bias. Hence, we recommend this approach for IPD-MA aimed at investigating subgroup effects.

### Conclusions

Our results confirm the benefit of appropriately specified one-stage meta-analysis methods to identify subgroup effects using individual participant data from multiple trials. Naive and centred IPD-MAs performed approximately equally well in terms of power and FPR, but since centred IPD-MA showed less estimate bias in the presence of ecological bias, we recommend the use of centred IPD-MA [5].

## Additional files

Additional file 1: Binary outcome (FPR and bias) (XLSX 14 kb)

Additional file 2: Continuous outcome (FPR and bias) (XLSX 14 kb)

Additional file 3: Large subgroup effect Binary outcome (power and bias) (XLSX 14 kb)

Additional file 4: Large subgroup effect Continuous outcome (power and bias) (XLSX 14 kb)

Additional file 5: Large subgroup effect Survival outcome (power and bias) (XLSX 14 kb)

Additional file 6: Medium subgroup effect Binary outcome (power and bias) (XLSX 14 kb)

Additional file 7: Medium subgroup effect Continuous outcome (power and bias) (XLSX 14 kb)

Additional file 8: Medium subgroup effect Survival outcome (power and bias) (XLSX 14 kb)

Additional file 9: Survival outcome (FPR and bias) (XLSX 14 kb)

## Data Availability

The simulated datasets used and analysis described in the current study are available from the corresponding author on reasonable request.

## Abbreviations

CPD-MA: Individual participant data meta-analysis
IPD: Individual participant data
MAPS: Per-subgroup meta-analysis
MA-IT: Meta-analysis of interaction terms
GLM: Generalized linear model
Cox: Cox proportional hazards
MLE: Maximum likelihood estimation
EB: Empirical Ayes
HKSJ: Shantung
FPR: False positive rate
